# Complete Genome Sequence of a Phenanthrene Degrader, *Burkholderia* sp. HB-1 (NBRC 110738)

**DOI:** 10.1128/genomeA.01283-15

**Published:** 2015-11-05

**Authors:** Yoshiyuki Ohtsubo, Azusa Moriya, Hiromi Kato, Natsumi Ogawa, Yuji Nagata, Masataka Tsuda

**Affiliations:** Department of Environmental Life Sciences, Graduate School of Life Sciences, Tohoku University, Sendai, Japan

## Abstract

The phenanthrene-degrading *Burkholderia* sp. HB-1 was isolated from a phenanthrene-enrichment culture seeded with a pristine farm soil sample. We report the complete genome sequence of HB-1, which has been deposited to the stock culture (NBRC 110738) at Biological Resource Center, National Institute of Technology and Evaluation (NITE), Tokyo, Japan. The genome of strain HB-1 comprises two circular chromosomes of 4.1 Mb and 3.1 Mb. The finishing was facilitated by the computational tools GenoFinisher, AceFileViewer, and ShortReadManager.

## GENOME ANNOUNCEMENT

A brown forest soil sample collected from pristine farmland of the Ehime Research Institute of Agriculture, Forestry, and Fisheries (Matsuyama, Japan) has been used for a number of microbial studies. Those include *in vivo* expression technology and signature-tagged mutagenesis studies of *Burkholderia multivorans* ATCC 17616 (1–3) to identify genes that are specifically induced and essential in the soil. This soil sample has also been used for a functional metagenomic study, in which the soil sample was artificially polluted with aromatic compounds to identify oxygenase genes (4). In addition, time-series responses of indigenous microbiomes to aromatic compounds revealed its robustness against chemical disturbance (5). This soil sample was used as a seed for a phenanthrene-enrichment culture in W medium, and repeated enrichment resulted in the domination of the genus *Burkholderia*, as revealed by 16S-rRNA gene sequencing. A portion of the culture was spread onto PCAT agar plates (6) with a semiselective medium for *Burkholderia* spp., and the phenanthrene-degrading *Burkholderia* sp. HB-1 was isolated. This strain grows on a phenanthrene-coated minimum agar plate and forms a clear zone; it degraded 2 mM phenanthrene within 2 days of incubation in the minimal liquid medium.

The strain HB-1 genome was sequenced using MiSeq systems (Illumina). A mate-pair (MP) library was constructed, and sequenced for 300 bp apiece from both ends to obtain 7.0-Mb pairs. Each pair of reads was processed by ShortReadManager to categorize it into three classes of MP, paired-end (PE), and single-end (SE) reads, while the read sequences were trimmed for adapters and inverted, as needed, to make the pairs inward-facing, A Perl script was used to merge PE reads that overlap for more than 30 bp completely.

We used Newbler version 3.0 to assemble those reads, and 3.3-Mb MP reads (623 Mb) and 2.1-Mb SE reads (396 Mb) were used for assembly. We obtained eight scaffolds and 88 contigs with a size larger than 500 bp and a coverage value greater than 20. The finishing was facilitated using GenoFinisher and AceFileViewer (7). Scaffold adjacencies were determined by GenoFinisher, except those among six scaffold ends, for which PCR experiments were conducted. All of the repeat-induced gaps were closed by using AceFilViewer. There were 45 gaps that arose due to lack of reads, and such gaps were closed by PCRs and sequencing of the PCR products.

The finished sequence was confirmed by FinishChecker. The complete sequence of the HB-1 genome comprised two circular chromosomes of 4,081,670 bp and 3,124,497 bp. The sequence was annotated by the NCBI Prokaryotic Genomes Automatic Annotation Pipeline (PGAP) and curated using GenomeMatcher (8). While referring to the annotation data obtained from the Microbial Genome Annotation Pipeline (http://www.migap.org), we corrected start codon positions and added genes that were missing in the PGAP annotation.

### Nucleotide sequence accession numbers.

The genome sequence of *Burkholderia* sp. HB-1 has been deposited in GenBank under the accession numbers CP012192 and CP012193.
